# Potential Mechanisms of Sensory Augmentation Systems on Human Balance Control

**DOI:** 10.3389/fneur.2018.00944

**Published:** 2018-11-12

**Authors:** Kathleen H. Sienko, Rachael D. Seidler, Wendy J. Carender, Adam D. Goodworth, Susan L. Whitney, Robert J. Peterka

**Affiliations:** ^1^Department of Mechanical Engineering, University of Michigan, Ann Arbor, MI, United States; ^2^Department of Applied Physiology and Kinesiology, University of Florida, Gainesville, FL, United States; ^3^Michigan Balance Vestibular Testing and Rehabilitation, Department of Otolaryngology, Michigan Medicine, Ann Arbor, MI, United States; ^4^Department of Rehabilitation Sciences, University of Hartford, Hartford, CT, United States; ^5^Departments of Physical Therapy and Otolaryngology, University of Pittsburgh, Pittsburgh, PA, United States; ^6^Department of Neurology, Oregon Health & Science University and National Center for Rehabilitative Auditory Research, VA Portland Health Care System, Portland, OR, United States

**Keywords:** biofeedback, sensory substitution, sensory augmentation, balance, sensory reweighting, balance prosthesis

## Abstract

Numerous studies have demonstrated the real-time use of visual, vibrotactile, auditory, and multimodal sensory augmentation technologies for reducing postural sway during static tasks and improving balance during dynamic tasks. The mechanism by which sensory augmentation information is processed and used by the CNS is not well understood. The dominant hypothesis, which has not been supported by rigorous experimental evidence, posits that observed reductions in postural sway are due to sensory reweighting: feedback of body motion provides the CNS with a correlate to the inputs from its intact sensory channels (e.g., vision, proprioception), so individuals receiving sensory augmentation learn to increasingly depend on these intact systems. Other possible mechanisms for observed postural sway reductions include: cognition (processing of sensory augmentation information is solely cognitive with no selective adjustment of sensory weights by the CNS), “sixth” sense (CNS interprets sensory augmentation information as a new and distinct sensory channel), context-specific adaptation (new sensorimotor program is developed through repeated interaction with the device and accessible only when the device is used), and combined volitional and non-volitional responses. This critical review summarizes the reported sensory augmentation findings spanning postural control models, clinical rehabilitation, laboratory-based real-time usage, and neuroimaging to critically evaluate each of the aforementioned mechanistic theories. Cognition and sensory re-weighting are identified as two mechanisms supported by the existing literature.

## Introduction

Active sensory augmentation (SA) for balance control is the focus of this critical review (1). We particularly highlight vibrotactile feedback but include other modalities of SA as well. We define SA as the delivery of additional sensory cues (e.g., via auditory, tactile, or visual modalities) that convey pertinent information about body orientation for balance. Passive forms of SA, such as mirrors, have been used during stroke rehabilitation (2, 3) and for treating phantom pain in amputees (4) since the 1990s. The first active form of SA was developed in the 1960s by Bach-y-Rita to provide vibrotactile cues to inform people with visual impairments about the location of an object (5). Shortly thereafter, the Naval Aerospace Medical Research Laboratory developed and piloted the Tactile Situation Awareness System (TSAS), an array of vibrotactile actuators worn on the torso, to augment a pilot's situational awareness and provide information about orientation and targeting (6). In the 1990's Wall adapted the TSAS concept for people with vestibular deficits (7) and Allum developed a multimodal feedback display for people with balance impairments (8).

SA for balance has been a focus of much research since the 2000's, likely influenced by increased availability of wearable technologies, especially compact, wireless, and accurate inertial measurement units. Various patient populations with primarily sensory-driven balance deficits have been included in research: people with vestibular loss, peripheral neuropathy, mild traumatic brain injury, and older adults, as well as people with stroke, Parkinson's disease, and ataxia.

**Figure 1:**
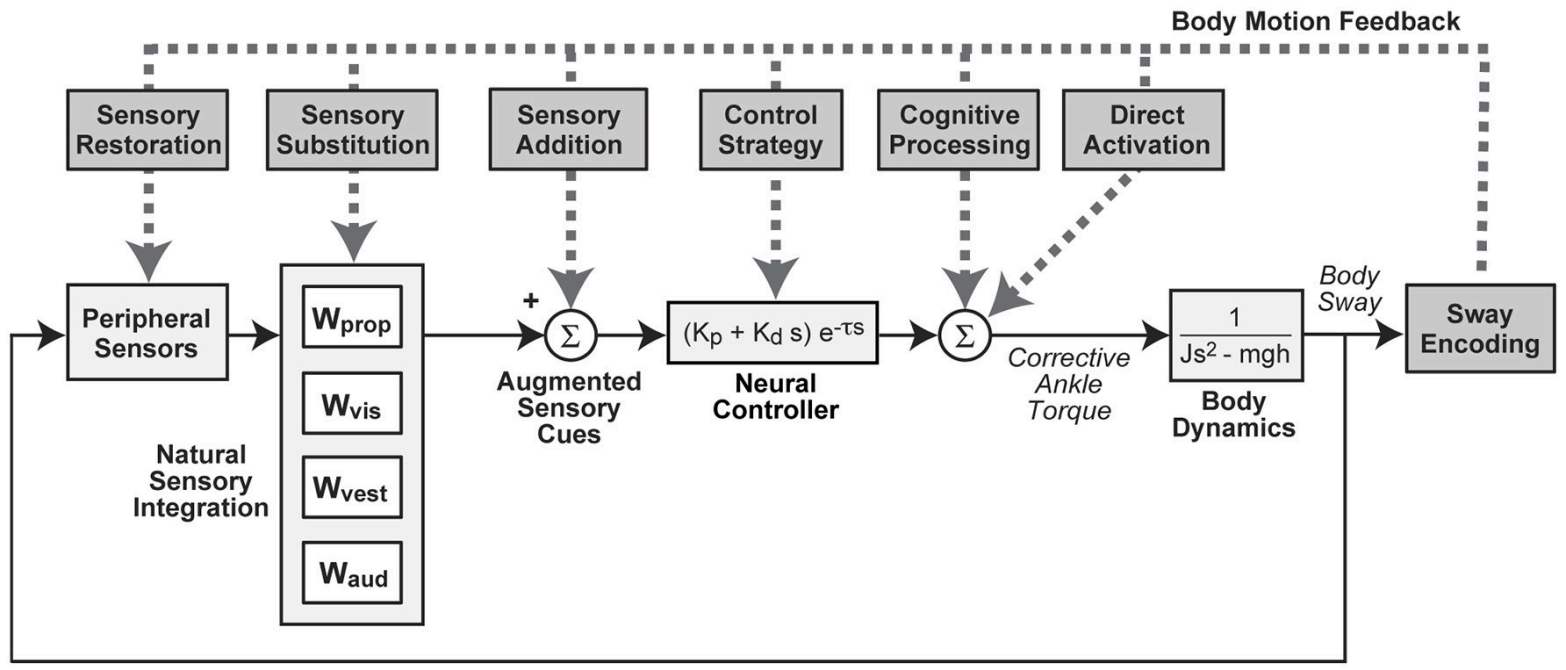
Block diagram representation of a simple feedback control model of balance showing potential modes of action by which measures of body sway could be used to improve balance control via sensory augmentation effects on different subsystems or by direct activation (e.g., functional electrical stimulation). Natural sensory integration is represented by a weighted combination of proprioceptive (W_prop_), visual (W_vis_), vestibular (W_vest_), and auditory (W_aud_) orientation information. Corrective torque generation is represented by a “neural controller” with stiffness (K_p_), damping (K_d_), and time delay (τ) parameters. Corrective torque is applied at ankle joint level to an inverted pendulum representation of the body with moment of inertia (J), mass (m), center of mass height (h). “s” is the Laplace variable and “g” is acceleration due to gravity.

Despite the recent interest in SA technologies, limited studies have investigated the underlying mechanisms of their effectiveness. However, several hypotheses are conceivable and a few have been historically proposed. These hypotheses can be conceptualized by considering how they influence various aspects of balance as represented by a simple model of balance control (Figure 1). We note that more than one mechanism could occur simultaneously.

“Sensory Restoration” refers to a device that fully restores missing sensory information. In this case, various methods for measuring balance function would show balance control behavior identical to that measured in subjects with normal sensory function. More likely the sensory restoration would be partial or limited. For example, for the foreseeable future a vestibular implant device at best will restore semicircular canal information, but not information from otolith organs (9). Examples of sensory restoration include retinal implants (10), cochlear implants (11), and vestibular implants (12).

“Sensory Substitution” refers to a device that acts through an alternative sensory modality (e.g., encoded using patterns of skin vibration) to convey the motion information that is related to that of a damaged sensory source. Ideally, this substituted information could be combined with other naturally available information and recognized by the brain as being equivalent to the damaged sensory source. If the information from the alternative sensory modality differs substantially from the damaged sensory information for which it is meant to substitute, the nervous system may not be able to combine it with other sensory sources in a natural way. In this case, it may be more appropriate to consider that the device is providing “Sensory Addition” (13, 14). Both sensory substitution and addition mechanisms can be thought of as augmenting balance control by making a “sixth sense” contribution to available sensory cues. Historically, sensory substitution and addition have been proposed as mechanisms when vibrotactile (15), auditory (16), or tactile (17) cues have been used to enhance visual inputs.

“Sensory Integration” refers to a mechanism that combines orientation information (often represented as a weighted combination) from various sources to serve as a basis for generating corrective actions that facilitate balance stabilization. Sensory restoration, substitution, and addition alter the available sensory information and are likely to have an impact on sensory integration via sensory reweighting. It has been posited that repeated exposure to an additional “channel” of body motion information provides the CNS with a correlate to the inputs from its intact sensory channels, promoting increased weighting of these intact channels and thereby promoting retentive (i.e., balance improvements are observed for the activities that were practiced/included in the training regime) and/or carryover (i.e., balance improvements are observed for activities that were not practiced/included in the training regime) effects once the additional channel of information is removed (18). Longer-term training with SA devices may affect sensory integration and context-specific adaptation by allowing time for the nervous system to develop optimal combinations/weights of sensory cues. Therefore, SA used during balance rehabilitation may lead to beneficial changes in sensory integration that are maintained even without the continued use of an SA device. Other SA benefits might arise from their influence on motor mechanisms. One could imagine that a device might motivate a change in “Control Strategy” that causes an individual to generate more or less corrective torque as a function of available sensory information. This could be represented by modification of neural control parameters where, for example, an increase in corrective torque generated per unit of body sway would cause a reduction in sway evoked by external disturbances even though sensory integration mechanisms remained unchanged. Temporary use of SA during balance rehabilitation may promote long-term changes in control strategy. Control strategy changes have been seen in subjects with Parkinson's Disease when receiving sensory cueing (19) and are likely influenced by individual motivation as well (20).

“Cognitive Processes” could have a role in explaining effects to the extent that subjects use conscious processing to generate voluntary actions to control balance. The TSAS for pilot situational awareness likely mediates cognitive processes and sensory addition (6). Finally, a device using functional electrical stimulation provides “Direct Activation” of muscles, thereby bypassing or partially bypassing natural sensory integration and muscle activation processes when they are not available or damaged (e.g., due to spinal cord injury) (21, 22). The aim of this critical review is to interpret aggregate findings in SA through the lens of several hypothesized mechanisms by first providing a brief overview of SA technologies for balance, then summarizing general outcomes for real-time use, balance rehabilitation, feedback modeling, and neuroimaging.

## Sensory augmentation technologies

**Figure 2:**
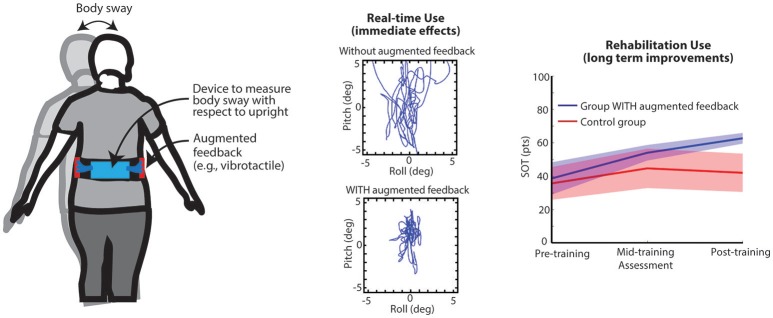
**Left**: Illustration of trunk-based IMU and vibrating actuators. **Center**: Bird's eye view of illustrative trunk sway data from a subject with cerebellar ataxia; top panel—real-time use without cues, bottom—real-time use with cues. **Right**: Pre-/per-/post-training computerized Dynamic Posturography SOT scores for two groups of older adults that performed balance training exercises 3x/week for 8 weeks in their homes either with or without (Control group) vibrotactile sensory augmentation (23).

Visual (e.g., mirrors) and haptic feedback provided through touch (e.g., walking aids such as canes, and real-time extrinsic feedback provided by a treating physical therapist via tactile cues and/or manual assistance to enhance movement, balance, and motor re-learning) are two of the most common forms of passive SA for balance applications. Modern technology-driven active SA devices typically couple inertial measurement units to estimate body kinematics and/or force plates or pressure-sensitive surfaces to estimate body kinetics with a wearable or off-body processor and a display (Figure 2). A variety of displays have been developed and reported in the literature to explore standing and gait-based feedback applications including arrays of vibrating actuators (7, 24), electrotactile arrays (15), televisions, or other various types of screens, headphones, or speakers (8, 25, 26), and combinations of multiple feedback modalities (27). Processors have included wearable computers, laptops or desktops, gaming systems (e.g., Nintendo Wii, Kinect), and smartphones (28). Specific feedback modalities may be preferential for certain patient populations based on compatibility with intact sensory systems (e.g., non-auditory information transfer for people with hearing loss). Likewise, for prolonged use, certain display modalities may pose challenges during activities of daily living. Presently, several devices (e.g., BalanceFreedom™ and SwayStar International™, and Vertiguard™) have been approved for use in Europe and South America. To date, a limited number of active SA devices have been approved by the FDA for use within the U.S. as a real-time balance or rehabilitation tool (e.g., Biodex Vibrotactile™ System). For the purposes of this critical review, we will explore potential general mechanisms of use as opposed to focusing on mechanisms associated with specific feedback modalities.

## Real-time use findings

Based on the published studies to date, the most likely dominant mechanism underlying balance benefits with real-time use of SA involves cognition; specifically, real-time SA cues are perceived, cognitively processed, and acted on based on the behavioral instructions assigned to the cues. The cognition hypothesis is supported by data that demonstrates that people's balance improves during the real-time use of SA cues compared to when no cues are provided, people's balance worsens when inaccurate cues are provided, people's balance is further improved when more information about body motion is provided, and people's temporal responses to the cues are on the order of several hundred milliseconds, which is consistent with response times associated with perceiving, processing, and responding to the cue.

To the extent that the effectiveness of an SA device depends on cognitive processing, sensory systems that naturally have good conscious representations, such as the auditory system, may be a better choice for delivering SA cues than sensory systems with poorer conscious representations. However, there is a tradeoff to be considered since the SA cues may interfere with the natural contribution to balance control provided by the sensory system used for SA. The auditory system is a good example since SA based on auditory feedback is commonly employed (8, 25–27, 29). Multiple studies have reported associations between hearing loss and increased fall risk in older adults (30, 31). The natural auditory contribution to spatial awareness likely involves unconcious processing of sound field cues that would likely be degraded by sound-based SA feedback.

The prominent literature base that supports this interpretation is a collection of numerous studies that have shown that people with vestibular deficits (28, 32) as well as older adults can use real-time SA cues to reduce sway when the stance position, support surface, or visual inputs are modified during standing balance tasks compared to conditions when cues regarding their body motion are not provided (27) (Figure 2). Young healthy adults (33), people with peripheral neuropathies (34), people with mild cognitive impairments (35), and stroke (36) have likewise shown reductions in postural sway related metrics compared with baseline. Real-time cues to inform modifications to gait have been shown to reduce M/L trunk tilt during paced heel-to-toe walking (37) and during narrow stance walking (38) in people with vestibular deficits. Young adults have also been shown to reduce their trunk sway and sway velocity when feedback was provided in the A/P or M/L directions during a variety of gait tasks (e.g., normal and tandem walking, climbing up and down stairs, walking over barriers), and gait velocity significantly increased when cues were provided (39). Older adults have been shown to increase their Dynamic Gait Index scores while using feedback (40). Young and older adults had reduced A/P and M/L tilt and A/P tilt velocity during normal walking (41). Cues have also been used to reduce knee adduction moments in people with knee osteoarthritis (42), alter plantar foot loading in people with stroke (43), and reduce gait asymmetry in people with cerebral palsy (44). When provided with combined auditory and vibrotactile SA, people with bilateral vestibular loss demonstrated decreased EMG amplitudes and less EMG background activity when standing on a compliant surface with their eyes closed (29).

When provided with erroneous cues, people with vestibular deficits initially demonstrate increased postural sway (24). However, it is believed that participants quickly ascertain that the erroneous cues conflict with other intact sensory inputs and participants ignore the inputs. This finding demonstrates that the cues are not simply serving as an alerting mechanism to prompt people to attend to their balance. However, it is possible that an alerting mechanism contributes to the observed improved postural control outcomes in the various real-time studies performed to date.

Continuous visual feedback has been shown to result in better performance than discrete visual or vibrotactile feedback, but some subjects reported dizziness when using continuous visual feedback (32). The improved results with the use of continuous feedback further support the cognitive hypothesis since people are provided with more complete information about their body motion and therefore are more likely to make more frequent and specific body corrections compared with discrete (less frequent, less information content) feedback.

Several studies have explored the effects of balance and gait parameters while simultaneously using an SA device and performing a secondary task; the findings partially support the cognition hypothesis because performance on the primary task generally declines when the secondary task is performed. Young subjects were able to use multimodal SA to reduce their trunk sway while walking and simultaneously counting backwards or carrying a tray of water (41). Older adults, however, were less responsive to the SA and subsequently less successful at reducing their trunk sway while concurrently performing a cognitive or motor task, potentially due to a lower residual processing capacity.

Interestingly, Lin et al. demonstrated that both younger and older adults had slower reaction times when performing an auditory reaction time test while using vibrotactile SA (45); however the older adults slowed disproportionally more on the reaction time task compared to the younger adults. There may have been more cognitive resources required to maintain balance with the dual task demands in the older subjects. However, balance differences based on kinetic measurements were not observed between persons with unilateral vestibular disorders and age-matched controls when tasked with using vibrotactile SA while simultaneously performing an auditory reaction time task on a computerized dynamic posturography platform (46). Both groups had slower reaction times when vibrotactile SA was provided, but the persons with vestibular loss were affected more profoundly.

Mechanical perturbations of the support surface have been employed to study how balance is affected by the use of vibrotactile SA feedback on the trunk. Significant reductions in falls during computerized dynamic posturography sensory organization test (SOT) conditions 5 and 6, which require more reliance on vestibular inputs, have been observed in people with severe vestibular deficits (47, 48). However, subjects with mild to moderate vestibular deficits did not fall as frequently as the severe group, and the number of their falls did not change significantly when they used feedback (47). Feedback may also promote faster recovery from discrete surface perturbations; specifically, peak tilt and the time to recover are decreased (47) (49). In a similar study examining the effects of vibrotactile feedback on the stepping responses of people with Parkinson's disease (PD) and age-matched controls, feedback cues did not affect the timing or the length of the steps, but it reduced trunk displacements prior to step initiation (50). Among young adults, older adults, people with bilateral vestibular deficits, and people with peripheral neuropathies, only older adults who exhibited slower stepping times during baseline trials showed significantly shorter stepping reaction times with versus without the feedback cue (51). These collective findings suggest that feedback is effective in reducing sway during normal stance and during recovery from perturbations, but not during the ballistic phase of a perturbation.

It should be noted that multiple studies have shown no reductions in sway during various gait tasks (8) and during non-challenging gait tasks (38) when vibrotactile SA feedback was provided on the trunk. Non-intended changes in gait patterns have also been observed, e.g., less natural gait patterns and altered segmental control strategies, although these changes may be due to inadequate training periods with the SA device. Multimodal SA may be more effective for improving gait performance compared to single sensory feedback in healthy older adults (26, 40, 52) and individuals post stroke (53–55).

Another potential mechanism that may contribute in a limited manner is the non-volitional response that has been observed when participants were presented with vibrotactile stimuli over the internal oblique and erector spinae locations; in addition to the small magnitude, the timing of the responses are likely too slow to have a significant impact on the initiation of postural corrections. Small, non-volitional sway responses to torso-based vibrotactile stimulation have been demonstrated when vibrations were applied over the internal oblique and erector spinae muscles. In these studies, participants were instructed to maintain an upright posture while standing with their arms at their sides. Movements on the order of approximately one degree were observed in the direction of the applied vibration (i.e., stimulation over the internal right oblique area resulted in a forward right movement), however, no motion was observed when stimuli were applied to the external oblique areas (56–60).

Vibration has also been used to improve signal detection in individual sensory channels. This particular use of vibration does not directly fit with our definition of SA because the vibration does not directly “convey pertinent information about body orientation for balance” but rather indirectly provides pertinent information by aiming to improve the detection of information obtained from existing peripheral receptors. This method of vibration has been termed stochastic resonance and relies on the theory that noise can improve the transmission and detection of information in some non-linear systems (61). Stochastic resonance applied as vibration to the bottom of the feet has been shown to reduce posture sway in quiet stance (61), one marker of improved feedback control. Others have applied the concept of stochastic resonance to activate the vestibular system via sub-threshold galvanic vestibular stimulation and also showed improvements in posture sway (62); these researchers also noted that a high noise level actually creates a distortion in vestibular feedback, increasing posture sway. Stochastic resonance could influence multiple mechanisms in the posture system. Clearly, the first mechanism is partial sensory restoration because the goal of stimulation is to improve the transmission of information from the peripheral sensors. With the improved transmission within one sensor, it is likely that sensory reweighting would take place because sensory reweighting is influenced by the accuracy and magnitude of peripheral feedback (63–65). The extent to which stochastic resonance impacts cognitive processes that contribute to balance is not well known.

## Rehabilitation using augmented sensory feedback

As a rehabilitation tool, SA can enrich and mimic the tactile and verbal cues provided by a physical therapist, thereby facilitating retraining of postural control for different patient populations, especially those with chronic imbalance (18). For SA to be an effective training tool, balance improvements achieved during the intervention should be retained after the feedback is removed and ideally carried over to other activities of daily living. The addition of SA to clinical and home exercise programs has the potential to provide the user with knowledge of results and maximize the participant's motivation and engagement (20).

Preliminary, small-scale studies showing balance improvements following training with SA versus. training alone suggest that augmentation facilitated training improves the utilization of available sensory cues via a sensory reweighting process. Sensory organization is an adaptive CNS regulated process, which enables a person to utilize the available, useful and accurate inputs to maintain balance in changing conditions or environments (66). Persons with compromised sensory systems (visual, vestibular, proprioceptive) may be able to use SA via a rehabilitation device to “upweight” (67) the available accurate information from the non-compromised system(s), or possibly enhance the “weakened signal” resulting in improved postural control. It appears that longer duration training with SA has better potential to enhance sensory reweighting (44). Persons with more severe sensory impairment have been found to benefit more from SA compared to those with moderate deficits, thus supporting the use of SA in acute stages of rehabilitation (47).

Several studies have demonstrated short-term retentive effects (24, 68–70) following short-term training with SA. However, many of the studies performed to date have been uncontrolled and therefore context-specific adaptation and/or habituation cannot be ruled out as a potential mechanism to explain the findings. In an uncontrolled study with Parkinson's population using Vertiguard, improvements in SOT scores were retained at three months post training and falls were reduced (71) following 10 training sessions within a two-week period. A limited number of controlled studies have examined retention and carryover effects following training with SA. In a randomized long-term home-based study in healthy older adults, participants trained for eight weeks using a smart phone balance trainer (28). The vibrotactile feedback group had greater improvements in SOT composite scores, which were maintained at six months, and both groups demonstrated improved vestibular reliance (23). In a recent clinical-based randomized preliminary study, a 6-week (18 sessions) vestibular rehabilitation program augmented with vibrotactile feedback was found to be beneficial for persons with unilateral vestibular disorders (72). The most significant finding was improved postural stability during balance exercises with head movements suggesting improved reliance on the available, but compromised, vestibular inputs. In a randomized control study, people with Parkinson's disease participated in 12 sessions of clinical balance training to compare the effects of virtual reality (VR) augmented balance training using a dynamic balance board (VR group) to conventional balance training (73). The VR group improved significantly on SOT condition 6 (unreliable vision and somatosensory inputs) immediately after training, however this finding was not significant at the four week follow-up suggesting limited retention effects.

Several studies have examined the incorporation of the Wii Fit balance board, which provides center of pressure (COP) information to the user, for balance training (74–82). Studies comparing the effectiveness of conventional physical therapy to Wii Fit balance training in older adults and persons with unilateral peripheral vestibular hypofunction found that balance training with virtual reality alone was not superior to traditional balance therapy (83, 84). Based on a recent systematic review, there is moderate evidence that visual feedback is beneficial in older adults with balance impairment (85). One study showed no overall benefit of balance training in healthy older adults when training was performed both with and without multimodal (vibrotactile, auditory, and visual) SA (86). Conversely, in a systematic review of frail older adults, both visual and auditory SA were noted to decrease sway although no large-scale randomized control trials were among the studies included (87).

Overall, there is moderate evidence to support the use of SA to improve postural control and gait during rehabilitation. In these balance-training scenarios, the real-time use of SA most likely involves cognition as described in the real-time use findings section above. Additionally, vibrotactile, visual, and/or auditory cues may simply alert users to momentarily attend to the balance or gait task at hand. There is limited evidence thus far for retention and/or carryover effects when the stimulus is removed following multiple training sessions. Longer use of SA has the potential to promote sensory reweighting and central compensation necessary to translate into longer-term retention and/or carryover, however, observed improvements in both control and intervention groups suggest that context-specific adaptation and/or habituation are also occurring.

## Sensory augmentation assessment using balance models

It can be difficult to ascertain causal relationships in standing balance because of complex time-delayed feedback interactions. To help interpret complex balance behavior, feedback models of posture control have been used for nearly two decades. To a remarkable extent, a relatively simple mathematical model of balance control, related to the model shown in Figure 1, has been shown to account very well for the dynamic characteristics of body sway evoked by continuously applied rotations of the stance surface or visual scene (88, 89). In the model, the body is represented by a single-segment inverted pendulum. Sensory integration is represented by a weighted summation of body orientation information derived from sensory cues; proprioception (signaling body sway relative to the surface), vision (signaling body sway relative to the visual scene), and vestibular (signaling body motion in space). Spatial cues derived from auditory information may also contribute to body orientation estimates used for balance control. Sensory-to-motor transformation is represented by a “neural controller” that generates time-delayed corrective ankle torque as a function of the integrated sensory information. The parameters of this model (mainly sensory weights, neural controller parameters, and time delay) can be estimated by optimally accounting for the experimentally observed relationship between a perturbing stimulus and the evoked sway response.

This simple model can serve as a reference for considering how SA devices affect different balance mechanisms. Although feedback modeling of SA for balance has not been widely used, three examples are presented below that provide insight into the mechanisms subjects use.

In one set of studies, vibrotactile feedback was provided to the torso of standing participants with vibration encoding a combination of body sway angle and sway velocity (13, 14). Body sway in healthy subjects was evoked in the sagittal plane with continuous pseudorandom surface tilts in eyes closed conditions, requiring participants to use both vestibular and proprioceptive feedback for balance. Experimental results were used to calculate frequency response functions that characterized the sensitivity and timing of sway responses across a wide range of frequencies (0.017–2.2 Hz). At low frequencies, vibrotactile feedback caused a reduction in sensitivity to the perturbing stimulus meaning that the subjects were better able to compensate for the perturbing influence of the stimulus and maintain a more vertical body orientation. But surprisingly, sensitivity to the stimulus slightly increased across higher frequencies. Additionally, vibrotactile feedback caused systematic changes in the timing of sway responses relative to the stimulus. To understand these results, the simple Figure 1 model was altered to investigate potential mechanisms of prosthesis action that could explain the experimental data. The investigators concluded that a “Sensory Addition” mechanism was best able to account for the results. Specifically, vibrotactile feedback provided a new sensory cue that summed with natural sensory cues, and did so without changing other characteristics of the balance control system. Additionally, the modeling results showed that the vibrotactile feedback was heavily low-pass filtered and time delayed (representing filtering of signal transduction across skin and/or CNS processing). Moreover, the model indicated that reliance on the vibrotactile feedback was highly dependent on the type of information encoded: participants relied upon the vibrotactile feedback more when it encoded body sway angle compared to sway velocity. A related study was able to predict how reliance changed with different combinations of angular position and velocity feedback by assuming participants optimally used augmented feedback to minimize a linear combination of sway angular position and jerk (the third derivative of displacement) (90).

A second set of studies demonstrated how the modeling results described above contributed to understanding the limited benefits obtained when the vibrotactile feedback was tested in subjects with bilaterally absent vestibular function (14, 91). Only limited improvements in balance were demonstrated and vestibular loss subjects were not able to maintain balance with eyes closed when the stance surface was sway-referenced (a condition that requires vestibular information). These experimental results rule out a “Sensory Substitution” mechanism, and are also consistent with the predictions of the “Sensory Addition” model developed from results in subjects with normal sensory function. Specifically, the model predicts unstable stance control if the only available cues about body sway in space are heavily filtered and time delayed.

A third study investigated “Sensory Restoration” provided via galvanic vestibular stimulation (GVS) in a subject with bilaterally absent vestibular function (92). The GVS delivered a current across electrodes applied to the mastoid processes behind the ears. In subjects with normal vestibular function GVS evokes sway in the frontal plane. If a vestibular loss subject retains sensitivity to GVS, the possibility exists that GVS feedback could partially restore a vestibular signal that encodes frontal plane body sway. When GVS was applied as a real-time function of frontal plane sway angle and sway velocity, application of system identification methods demonstrated that GVS feedback caused a reduction in sensitivity to a surface-tilt perturbation performed with eyes closed, consistent with a partial restoration of vestibular information for balance control. Since GVS is considered to have its primary net influence on head velocity information encoded by the semicircular canals, experiments using GVS feedback may be directly relevant to predicting changes in balance control afforded by future vestibular prostheses that target electrical activation of the canals.

It is important to note that the studies described above examined only short-term applications of SA devices. It is entirely possible that sensorimotor learning mechanisms could improve effectiveness over time.

## Neuroimaging of sensory augmentation

Functional neuroimaging has provided insight into the neural control of movement in human subjects, and how control networks change in response to a variety of interventions and rehabilitation training programs. Not as much progress has been made in understanding the functional brain networks which contribute to static and dynamic balance, however, because most neuroimaging technologies require subjects to lay supine during brain scanning. Moreover, head movements can result in motion artifacts for neuroimaging data. Therefore, most neuroimaging studies of vestibular function have been conducted while participants passively receive vestibular stimulation laying supine and still.

Given the challenges of using neuroimaging tools to study balance control, it is perhaps not surprising that only a few studies have investigated the neural correlates of SA-induced improvements in balance. One exception is a line of work from Wildenberg et al. (93–95), which extends work by Bach-y-Rita using electrotactile tongue stimulation to convey relative head position information [cf. (96)]. This work provides some insight into the underlying mechanisms of at least one form of SA. Initial studies with this device were focused on real-time benefits; it should be noted, however, that the neuroimaging work has all been conducted using a rehabilitation approach. That is, functional neuroimaging was conducted before and after multiple sessions of SA, and, because participants were supine and still during the imaging, the SA system was not used in the scanner.

## Brain changes associated with rehabilitation-based sensory augmentation

Wildenberg and colleagues conducted neuroimaging before and after several sessions in which participants wore an accelerometer on the head and had real-time head position information conveyed to them via electrotactile tongue stimulation. This technique has been shown to improve both objective and subjective measures of gait and balance both during real-time use and also extending beyond the stimulation sessions, in both healthy individuals and those with vestibular or visual deficits (97–101). The initial hypothesis was that this particular form of SA was effective due to “spillover” of neural activity from the tongue afferent pathway to the vestibular nuclei, adjacently located in the brainstem (102). To evaluate this hypothesis, Wildenberg et al. (94, 102) acquired functional MRI while balance impaired subjects passively viewed either static or expanding and retracting visual flow both before and after nine sessions of quiet stance coupled with tongue electrotactile SA. The subjects showed greater activity in response to visual flow patterns in brain regions that process visual motion including in the occipital lobe and cerebellar vermis. Interestingly, after training with this SA, postural sway was less susceptible to disturbance when subjects viewed optic flow stimuli, and the over-activation of visual motion processing regions was reduced. These findings support the notion that balance training coupled with SA acted via a sensory reweighting mechanism to reduce reliance on visual cues in balance impaired subjects who were initially overly reliant on visual inputs. There was also increased activity post training in the brainstem, supporting the possibility that activity in the tongue afferent pathway may have spread to vestibular brainstem regions as well.

These authors have also shown that tongue electrotactile stimulation aids balance even when the stimulation carries no information about body position. That is, the pattern of stimulation does not have to be coupled with head motion in order to result in decreased postural sway (93, 102). Stimulation that is not coupled to body position does not meet our definition of SA; we present the findings here however because the studies are direct follow ups to those described in the preceding paragraph. To more precisely investigate the brainstem changes occurring with stimulation, Wildenberg et al. (93) conducted a high resolution MRI study of changes in brainstem activity from 19 sessions of tongue electrotactile stimulation. Prior to the intervention, optic flow stimuli produced activation in several brainstem regions including the trigeminal and vestibular nuclei as well as the superior colliculus. After the stimulation sessions, there was increased activation in the pons. The authors suggested that this increased activity in the pons was in the trigeminal nucleus, part of the tongue afferent pathway. They further hypothesized that spread of excitation from this region to the vestibular nucleus resulted in enhanced balance.

A recent study that evaluated vibrotactile feedback delivered to the torso as a rehabilitation balance aid, coupled with in-home balance training, found evidence that this form of SA also affected sensory reweighting. The group of healthy older adults that trained with SA showed a greater increase in reliance on vestibular inputs from pre to post training than the group that performed balance exercises alone (23). A subset of the subjects underwent fMRI scans pre and post training while receiving vestibular stimulation with a pneumatically powered tapper device that elicited ocular vestibular evoked myogenic potentials (oVEMPs) and activation in vestibular cortex (103). The fMRI results showed increased activity in brain regions which process somatosensory, visual, and vestibular inputs following training suggesting that SA with balance training alters sensory processing and integration. Further research with additional subjects is required to determine whether and how these brain changes relate to functional balance improvements, how long the brain changes are retained, and whether they differ between participants that receive balance training alone and those that receive training plus SA. It is interesting that both the brain and behavioral changes suggest shifts in sensory reliance and integration with training; participants increased their reliance upon vestibular inputs for balance following training with vibrotactile SA. The work discussed in this section on rehabilitation-based SA supports sensory reweighting as an underlying proposed mechanism. The activation “spillover” described above could play a role in this reweighting, or the brain may instead rely upon this as a “sixth sense” type of proposed mechanism. Regardless, it appears that real-time SA may be effective by eliciting new cognitive strategies, whereas rehabilitation-based SA appears to result in sensory reweighting.

## Summary

Current SA applications impact balance control through a variety of mechanisms. Because each mechanism has its own characteristic features, it is worth considering which mechanism applies to a given application in order to anticipate its limitations and potential benefits. Real-time feedback via a sensory restoration mechanism likely has the greatest potential for restoring normal balance function since the sensory information flows through neural channels specifically involved in natural balance control. SA using future vestibular implants, galvanic vestibular stimulation, and foot vibrations to enhance proprioception are sensory restoration applications. For real-time use of SA, results favor a cognitive or sensory addition mechanism, but not a sensory substitution mechanism since substitution implies an equivalency between information provided by the SA and natural sensory systems. A cognitive feedback loop that relies on voluntary commands to control balance could have similar functional characteristics to a sensory addition mechanism (e.g., both having long time delays), but reliance on cognitive control would be inferior to sensory addition as a balance aid due to a need for constant attentiveness. Studies that apply long-term SA are needed to see if a balance aid with features of a sensory addition mechanism can evolve through motor learning to behave as a sensory substitution mechanism where the augmented sensory information is used in a manner that is essentially indistinguishable from natural sensory feedback. Prolonged balance training with SA would ideally improve balance after the augmentation is removed. However, there are mixed results supporting this positive retention and carryover. When retention and carryover are found, evidence supports the notion that SA altered sensory integration via a sensory reweighting mechanism. Finally, application of system identification methods employing model-based interpretation of experimental results can provide detailed quantitative measures of the balance control system to assess the effectiveness of SA technologies and rehabilitation strategies.

## Author contributions

KS, RS, WC, AG, SW, and RP wrote the manuscript. AG and RP created the figures.

### Conflict of interest statement

The authors declare that the research was conducted in the absence of any commercial or financial relationships that could be construed as a potential conflict of interest.
